# Prevalence and risk factors of lactic acidosis in children with acute moderate and severe asthma, a prospective observational study

**DOI:** 10.1007/s00431-020-03834-x

**Published:** 2020-10-22

**Authors:** Marta Ruman-Colombier, Isabelle Rochat Guignard, Ermindo R. Di Paolo, Mario Gehri, Jean-Yves Pauchard

**Affiliations:** 1 Children’s Hospital, Service of Paediatrics, Department of Woman, Mother, Child, Lausanne University Hospital, University of Lausanne, Chemin de Montétan 16, 1004 Lausanne, Switzerland; 2 Respiratory Unit, Service of Paediatrics, Department of Woman, Mother, Child, Lausanne University Hospital, University of Lausanne, Rue du Bugnon 46, 1011 Lausanne, Switzerland; 3 Service of Pharmacy, Lausanne University Hospital, University of Lausanne, Rue du Bugnon 46, 1011 Lausanne, Switzerland

**Keywords:** Lactic acidosis, Salbutamol, Asthma, Paediatrics

## Abstract

Lactic acidosis is a common complication of status asthmaticus in adults. However, data is sparse in children. The aim of this study was to describe the prevalence and risk factors for lactic acidosis in children hospitalised for acute moderate or severe asthma. A total of 154 children 2–17 years of age were enrolled in a prospective observational study conducted in a tertiary hospital. All had capillary blood gas assessment 4 h after the first dose of salbutamol in hospital. The primary endpoint was the prevalence of lactic acidosis. Potential contributing factors such as age, sex, BMI, initial degree of asthma severity, type of salbutamol administration (nebuliser or inhaler), steroids, ipratropium bromide, and glucose-containing maintenance fluid represented secondary endpoints. All in all, 87% of patients had hyperlactatemia (lactate concentration > 2.2 mmol/l). Lactic acidosis (lactate concentration > 5 mmol/l and anion gap ≥ 16 mmol/l) was observed in 26%. In multivariate analysis, age more than 6 years (OR = 2.8, 95% CI 1.2–6.6), glycemia above 11 mmol/l (OR = 3.2 95% CI 1.4–7.4), and salbutamol administered by nebuliser (OR = 10, 95% CI 2.7–47) were identified as risk factors for lactic acidosis in children with moderate or severe asthma.

*Conclusion*: Lactic acidosis is a frequent and early complication of acute moderate or severe asthma in children.

**Table:** 

**What is Known:** *• Lactic acidosis during acute asthma is associated with b2-mimetics administration.* *• Salbutamol-related lactic acidosis is self-limited but important to recognise, as compensatory hyperventilation of lactic acidosis can be mistaken for respiratory worsening and lead to inappropriate supplemental bronchodilator administration.* **What is New:** *• Lactic acidosis is a frequent complication of acute asthma in the paediatric population.* *• Age older than 6 years, hyperglycaemia, and nebulised salbutamol are risk factors for lactic acidosis during asthma.*

**What is Known:**

*• Lactic acidosis during acute asthma is associated with b2-mimetics administration.*

*• Salbutamol-related lactic acidosis is self-limited but important to recognise, as compensatory hyperventilation of lactic acidosis can be mistaken for respiratory worsening and lead to inappropriate supplemental bronchodilator administration.*

**What is New:**

*• Lactic acidosis is a frequent complication of acute asthma in the paediatric population.*

*• Age older than 6 years, hyperglycaemia, and nebulised salbutamol are risk factors for lactic acidosis during asthma.*

## Introduction

Asthma is characterised by chronic airway inflammation and hyperresponsiveness. During an acute exacerbation, inhomogeneous airway narrowing and obstruction lead to hypoxemia. Compensatory hyperventilation mediated by pulmonary mechanoreceptors conducts to respiratory alkalosis, the most frequent acid-base disturbance observed in acute asthma. If not treated properly, progressive respiratory insufficiency may occur with hypercapnia and respiratory acidosis [1].

Lactic acidosis is another blood gas alteration observed during moderate or severe asthma. Multiple mechanisms have been proposed. Some have evoked reduced tissue perfusion or overuse of respiratory muscles under hypoxic conditions [2]. Others have suggested that b2-adrenergic agents like bronchodilators used to treat asthma lead to increased gluconeogenesis, glycogenolysis, glycolysis, and lipolysis, cumulating in lactic acid production [3–6]. Depending on the mechanism of lactate formation, two types of lactic acidosis exist that can be distinguished calculating the lactate to pyruvate ratio (L/P). Type A (L/P ratio < 25/1) is related to impaired oxygenation, and type B (L/P > 25/1) is caused by excessive b2-receptor stimulation [6].

Lactic acidosis is now a well-known complication of status asthmaticus in adults. Case reports and some retrospective and rare prospective studies describe a transient lactic acidosis as a side effect of high doses b2-agonists used in acute asthma treatment. However, data is rare in children [7].

Even if lactic acidosis during asthma is a self-limited condition, it has an impact on assessment and management of respiratory distress. Compensatory hyperventilation of lactic acidosis is often mistaken as a sign of respiratory worsening and leads to inappropriate escalation of bronchodilator therapy, increasing morbidity and mortality [8, 9].

The aim of our study is to describe the prevalence and risk factors contributing to lactic acidosis in children treated with salbutamol for moderate or severe acute asthma.

## Materials and methods

### Study design

This prospective observational monocentric prevalence study was conducted from May 01, 2017, to April 30, 2019, in a tertiary care children’s hospital.

### Patients

Children and adolescents 2 to 17 years of age hospitalised for acute moderate or severe asthma were eligible for the study. At our institution, patients requiring inhaled b2-agonists minimum every 2 h fill the indications to be admitted, and an initial degree of asthma severity based on PRAM score is part of the clinical evaluation [10]. Exclusion criteria were as follows: parent’s refusal, metabolic disorder, shock, sepsis, renal or hepatic insufficiency, diabetes mellitus, and cancer.

### Data collection

Basic clinical data included age; body mass index (BMI); sex; initial degree of asthma severity; dose and type of salbutamol administration, of oral or intravenous steroids, and of inhaled ipratropium bromide; and type of maintenance intravenous fluid. Hypoxemia (defined as SpO2 < 92%) was rigorously assessed and treated. Salbutamol was administered by using a metered dose inhaler (pMDI) (through a valved holding chamber with a mouthpiece or a mask, when necessary, AeroChamber Plus Flow Vu®, 1push = 100 cmg of salbutamol) or nebuliser (aerosol solution of 5 mg of salbutamol in 5 ml of normal saline solution NaCl 0.9%). Three types of maintenance fluid were used: 91% of G10% and 9% of NaCl 10% mix, 91% of G5% and 9% of NaCl 10% mix, or normal saline solution (NaCl 0.9%). Capillary blood sample was drawn 4 h after the first administration of salbutamol in hospital, or in case of secondary appearance of tachypnoea or worsening of the respiratory status during hospitalisation. Oxygen saturation was measured by pulse oximetry before blood extraction. Blood gas assessment including the measurement of pH, pCO_2_, HCO_3_, base excess (BE), and glucose and lactate concentrations was performed on a RAPIDPoint500, Siemens, gasometer. One millilitre of blood was used for analysis. Hyperlactatemia was defined as lactate concentration > 2.2 mmol/l. Lactic acidosis was defined as lactate > 5 mmol/l and anion gap (AG) ≥ 16mmol/l (AG = Na + K-HC0_3_-Cl), non-compensated lactic acidosis as pH < 7.35, and lactate concentration as > 5 mmol/l. Compensated lactic acidosis represented lactate concentration > 5 mmol/l, pH ≥ 7.35, and pCO2 < 35 mmHg. Hyperglycaemia was characterised as glucose > 11 mmol/l.

### Outcomes

The primary outcome was the prevalence of lactic acidosis. Secondary outcomes included other contributing factors like age, sex, BMI, initial degree of severity, salbutamol administered by inhaler or by nebuliser, steroids, ipratropium bromide, and glucose-containing maintenance fluid.

### Ethical considerations

Patients older than 11 years and parents or legal guardians were informed orally and in writing about the research project by one of the doctors working in the paediatric emergency room during admission to the hospital. Adolescents ≥ 14 years and their parents or legal guardians provided informed consent. The study was approved by the institutional ethics committee (Swiss Ethics, protocol number 2016-01320).

### Statistical analysis

Categorical data were described as absolute counts. Percentages and continuous data we described as means and standard deviations (SD). The Mann–Whitney *U* test was used to compare the means. We firstly realised simple logistic regression and calculated odd ratio for all potential risk factors. All variables potentially able to influence lactic acidosis (*p* < 0.2) were used as covariates in multiple logistic regression. Pearson’s correlation coefficient was used to measure statistical relationship between the levels of lactates and doses of salbutamol. All statistical analysis was performed with the Epi Info version 7.2.3.1 software (Centres for Disease Control and Prevention).

## Results

**Fig. 1:**
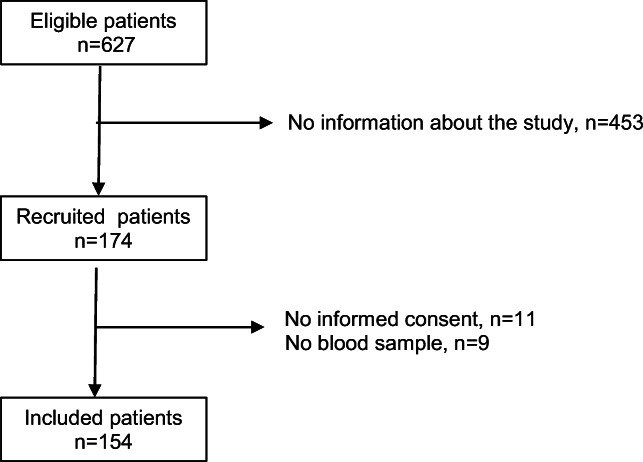
Flow chart of study population

Among 627 patients from 2 to 17 years of age hospitalised for moderate or severe asthma, 174 received information about the study. Eleven did not provided informed consent, and 9 did not have blood sample for technical problem. Finally, 154 patients were included in the study (Fig. 1)

Among 154 patients, 99 (64%) were aged from 2 to 6 years and 55 (36%) were more than 6. A total of 13% were obese (*P* > 97‰). Sex ratio was 1.92 male to female. Forty-three percent of episodes were categorised as severe (PRAM > 8) and 57% as moderate (PRAM 4–8). Salbutamol was administered by pMDI with holding chamber alone in 30% of cases (46/154) or by nebuliser (alone or associated with pMDI administration) in 70% (108/154) (Table 1). Mean overall dose of salbutamol administered during the first 4 h after hospital admission was 12 ± 10 mg. The mean dose was similar in older (> 6 years of age) and younger groups (≤ 6 years of age), 12.3 ± 10.5 mg and 11.8 ± 9.8 mg respectively (*p* = 0.59). Patients with severe asthma received higher doses of salbutamol (mean 19 ± 10.1 mg) compared to patients with moderate asthma (mean 6.67 ± 5.7 mg), *p* < 0.0005. When delivered by nebulisation (1 nebulisation = 5 mg of salbutamol), the mean dose of salbutamol was 16 ± 9.3 mg versus 2.4 ± 1.2 mg when delivered by inhaler (1 push = 100 cmg), *p* < 0.0005.

**Table 1:** Characteristics of study population

Characteristics	*n* = 154	%
Age ≥ 6 years of age	55	36
Female sex	51	33
Severe asthma (PRAM 8–12)	66	43
Moderate asthma (PRAM 4–7)	88	57
PICU admission	0	0
Obesity	20	13
Salbutamol administered by inhaler	46	30
Salbutamol administered by nebuliser	108	70
Intravenous salbutamol	0	0
Aminophylline	0	0
Corticoids	146	95
Ipratropium bromide	23	15
Glucose-containing maintenance fluid	30	19
Hyperlactatemia > 2.2 mmol/l	134	87
Hyperlactatemia > 5 mmol/l	40	26
Non-compensated lactic acidosis (lactate > 5 mmol/l and pH < 7.35 mmHg)	6	4
Compensated lactic acidosis (lactate > 5, pH > 7.35, pCO2 < 35 mmHg)	34	22
Hyperglycaemia	56	36

Almost all patients (95%) received corticosteroids with a mean dose of 1.85 ± 0.75 mg/kg. Corticosteroids were mainly administered orally (88%). Twenty-three of 154 patients (15%) received ipratropium bromide. Nineteen percent were perfused with glucose-containing maintenance fluid (91% of G10% and 9% of NaCl 10% mix or 91% of G5% and 9% of NaCl 10%) (Table 1).

All patients had capillary blood gas analysis 4 h after the first dose of salbutamol administration. Secondary appearance of tachypnoea or worsening of the respiratory status motivated a second blood gas analysis in 13 patients.

Most of the patients (87%) had mild hyperlactatemia (lactate > 2.2 mmol/l). All of patients with lactate concentration > 5 mmol/l (26%) had lactic acidosis (lactate > 5 mmol/l and augmented anion gap AG ≥ 16 mmol/l). Thirty-four (22%) presented compensated lactic acidosis (lactate > 5 mmol/l, pH ≥ 7.35, and pCO2 < 35 mmHg). Only 6 (4%) had pH < 7.35. None had hypercapnia (pCO2 > 40 mmHg) (Table 1).

In univariate analyses, a significant correlation was found between lactic acidosis and female sex (OR = 2, 95% CI 1–4.2), as well as between lactic acidosis and severe asthma (OR = 4, 95% CI 1.9–8.6). When adjusted on potential confusing factors in multivariate analysis, salbutamol administered by nebuliser (aOR = 10, 95% CI 2.7–47), age older than 6 years (aOR = 2.8, 95% CI 1.2–6.6), and hyperglycaemia (aOR = 3.2 95% CI 1.4–7.4) were related to increased risk of lactic acidosis (Table 2).

**Table 2:** Risk factors of lactic acidosis

Risk factors of lactic acidosis	Number of patients (%)		Univariate analysis unadjusted OR [95% CI]	Multivariate analysis adjusted OR [95% CI]
Lactic acidosis (yes/non)	*n* = 40	*n* = 114		
Female sex	18 (45)	33 (29)	2 [1–4.2]	1.9 [0.8–4.4]
Age ≥ 6 years of age	19 (48)	36 (32)	1.9 [1–4.1]	2.8* [1.2–6.6]
Severe asthma	27 (68)	39 (34)	4 [1.9–8.6]	2 [0.8–5.2]
Obesity	8 (20)	12 (11)	2.1 [0.8–5.7]	2.5 [0.8–8.1]
Hyperglycemia (> 11 mmol/l)	24 (60)	32 (28)	3.8 [1.8–8.2]	3.2* [1.4–7.4]
Glucose-containing maintenance fluid	8 (20)	22 (19)	1.05 [0.4–2.6]	
Corticoids	41 (100)	106 (95)		
Ipratropium bromure	8 (20)	15 (13)	1.7 [0.6–4.3]	
Salbutamol administered by nebulisation	38 (95)	70 (61)	11.9 [2.7–52]	10* [2.3–47]

*OR*, odds ratio; *CI*, confidence interval

Number of patients was expressed in absolute number and in percentage (%)

All variables associated with *p* < 0.2 were included in the multivariate analysis

Significative findings were marked with an asterisk (*p < 0.05)

**Fig. 2:**
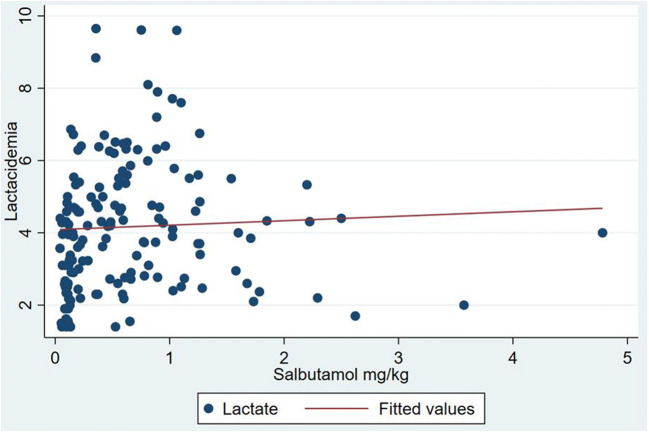
Relation between lactate levels and doses of salbutamol

Lactic acidosis was observed even with low doses of salbutamol (< 0.5 mg/kg). No correlation was found between lactate levels and salbutamol doses (Pearson’s *r* = 0.17 (SE = 0.24)) (Fig. 2).

## Discussion

Although lactic acidosis is a well-known metabolic disturbance of asthma in adults, data are rare in the paediatric population. To our knowledge, this is the first large prospective study describing prevalence and risk factors for lactic acidosis in children with acute moderate or severe asthma.

A total of 87% (134/154) of our patients had increased blood lactate concentration (lactate > 2.2 mmol/l) 4 h after the first salbutamol dose in hospital. Our findings compare to those of Meert who recorded 83% (62/105) of children with mild hyperlactatemia (lactate> 2.2 mmol/l) during status asthmaticus [5]. Lower prevalence (71%) was found in a retrospective study of 75 children, but the time of lactatemia assessment was not specified [11]. In adults, the prevalence of mild hyperlactatemia (lactate > 2 mmol/l) ranges from 59 in 29 patients with severe asthma 4 to 6 h after therapy beginning [12] to 69.2% at an earlier time point for lactate measurement (about 1 h 25 min after albuterol treatment beginning) [13].

In paediatric case reports, lactate concentration in asthma-related lactic acidosis ranged from 5.9 to 9.2 mmol/l [8]. Koul documented lactic acidosis with a peak lactate range (5.2–13 mmol/l) 2–8 h after the beginning of aerosol therapy in 4 children 11 to 17 years of age. In our study, lactatemia varied from 1.4 to 9.66 mmol/l 4 h after salbutamol administration, with 26% of patients presenting respiratory-compensated lactic acidosis (lactate > 5 mmol/l and AG ≥ 16 mmol/l, pCO2 < 35 mmHg), consistent with other observations [14]. In a retrospective study of 75 children with acute asthma, metabolic acidosis (pH < 7.35 and BE < − 7) was found in 21% of patients [11]. Available lactate level was > 5 mmol/l in 22% of children. Likewise, Meert identified 28% of 53 patients with metabolic acidosis (pH < 7.35, PCO_2_ < 35 mmHg, and BE < − 7 mmol/l) during acute asthma, with lactate assessment from 7.2 to 9.3 mmol/l 8 to 24 h after admission [4]. Lastly, lactic acidosis with or without respiratory compensation was identified in 47 of 105 (45%) children with acute asthma [5].

In critically ill paediatric patients with asthma, acidosis (pH < 7.35) was found in 45% of patients admitted to the intensive care unit (ICU). Only one had metabolic acidosis with hyperlactatemia (4.6 mmol/l 6 h after ICU admission). All the others had acidosis from respiratory origin. However, in these patients, the blood gas determination was realised quite early, within 2 h after emergency room admission, and the dose of salbutamol received was not specified. Yousef did not find metabolic acidosis in eight other episodes of severe respiratory failure attributable to asthma and suggested that lactic acidosis during asthma is not underestimated and children may be more resistant than adults to the development of this complication. He implied that metabolic acidosis reported in previous studies could be rather related to ketosis following suboptimal hydration and caloric management [15]. We cannot support this hypothesis because all our patients unable to feed or to drink received intravenous glucose perfusion.

Lactic acidosis implies two mechanisms. Type A is associated with impaired oxygen delivery and/or hypotension. Type B implies underlying disease (liver or renal insufficiency, diabetes mellitus, or cancer), drugs (such as b2-agonists), or inborn errors of metabolism [16]. None of our patients had known chronic underlying disease. Many authors thought lactic acidosis during asthma to be type B [4–6]. Exposition to high doses of bronchodilator-type salbutamol induces hyperadrenergic state and leads to increased lactate production. Presence of lactic acidosis in patients receiving b-2 agonist therapy under optimal oxygenation or artificial ventilation supports this hypothesis [17]. On the biological level, types A and B can be distinguished by the L/P ratio (L/P < 25/1 versus L/ P> 25/1, respectively). Meert calculated the L/P ratio and concluded that type B lactic acidosis is the most frequent in asthma [5]. Even if hyperlactatemia has been described as a marker of mortality in critically ill patients [18], type B lactic acidosis is a self-limiting condition, and no fatal case has been described in children. The spontaneous resolution with decreasing doses or discontinuation of bronchodilator therapy is a rule [4, 5]. In our study, we did not perform pyruvate assessment for technical reasons and thus could not ascertain the mechanism of lactic acidosis precisely, but we highly suggest type B because none was hypoxemic at the time of lactate assessment and a favourable evolution was observed for all.

In our patients, mean total dose of salbutamol delivered by nebulisation (16 ± 9.3 mg) was almost seven times higher compared to the mean dose delivered by inhalator (2.4 ± 1.2 mg). Higher doses could explain the greater risk of lactic acidosis if salbutamol is administered by nebulisation (RR = 10, 95% CI 2.3–47). According the Cochrane database, other side effects of salbutamol such as increased pulse rate were lower for pMDI in children (mean difference − 5% baseline, 95% CI − 8 to − 2%), as was the risk of developing tremor (RR = 0.64; 95% CI 0.44 to 0.95) [19]. On the other hand, in the paediatric population, only 1–10% nebulised salbutamol reaches the inferior respiratory tract [20–23]. Previous study by Wildhaber has shown equivalent percentages of total lung deposition of radiolabeled salbutamol aerosolised by using either a nebuliser or a pMDI with holding chamber (9.6% and 11% for inhaled and nebulised respectively in children > 4 years of age and 5.4% for both in children < 4 years of age) [22]. In a more recent study, it was shown that the amount of drug delivered from pMDI was higher, ranging from 18.1 to 22.5% in young children (3–5 years of age) [22, 23] and from 35.4 to 54.9% in older children (5–17 years of age) [24]. The authors concluded that most children from 5 years of age could obtain lung deposition of more than 30% using a tidal breathing technique with a pMDI. All our patients receiving salbutamol by inhaler via pMDI used this inhalation technique. We did not find a correlation between lactate levels and doses of salbutamol. The statistical power of a dose correlation would be probably reduced by a large proportion of children with mild hyperlactatemia (2.2–5 mmol/l).

Intravenous, oral, and inhaled salbutamol raise glycogenolysis resulting in hyperglycaemia. Concurrent use of corticosteroids may exacerbate blood glucose level [6]. In our study, almost all patients received concomitant steroids (95%), and 36% of them had hyperglycaemia (glucose > 11 mmol/l). b2-agonist drugs have two main actions. Firstly, b2-adrenergic receptor stimulation increases glycogenolysis, neoglucogenesis, and glycolysis leading to transformation of glucose to glucose 6-phosphate and then to pyruvate. Secondly, b2-agonists enhance lipolysis. Free acids inhibit pyruvate dehydrogenase, an enzyme which normally allows pyruvate to enter the Krebs cycle. In this way, pyruvate to lactate formation is promoted [25]. In our study, we show that hyperglycaemia raises the risk of lactic acidosis during asthma (aOR = 3.2 95% CI 1.4–7.4). On the intracellular level, the rise in glucose blood level via bronchodilator-mediated glycolysis provides more substrate for lactate production. In patients with severe asthma in ICU, serum glucose was measured. Even if 88% of them had hyperglycaemia (> 6.8 mmol/l), the relationship between hyperglycaemia and lactate concentration could not be proved [4]. Our study showed that children aged more than 6 have almost three times more risk to develop lactic acidosis during asthma (aOR = 2.8, 95% CI 1.2–6.6), despite the fact that the mean dose of salbutamol was the same in both age groups (mean of 11.8 mg, SD 9.8 for < 6 years versus 12.3 mg, SD 10.5 for > 6 years). It could be related to the fact that younger children dispose less glucose resources and in consequence less substrate for lactate production.

We identified other parameters like female sex, severe asthma, and obesity as independent risk factors for lactic acidosis. Even if they could not be confirmed in multivariate analysis, they need to be put forward as our study is the first one to try to identify potential risk factors of lactic acidosis in children with asthma.

Our study has some limits. Firstly, only 25% of potential patients with moderate or severe asthma seen in the emergency room were included, which is a major selection bias. Secondly, we performed only capillary lactate assessment. However, prior research suggests that capillary lactate value accurately reflects arterial lactate [26]. Moreover, this technique is quicker and easier to perform, especially in children. Another limitation is the lack of initial lactate level. It would be interesting to compare the lactate level upon arrival and 4 h later. Finally, the relationship between steroid therapy and lactic acidosis could not be investigated because almost all patient received corticosteroids.

## Conclusion

Lactic acidosis is a frequent and early (H4) complication of asthma observed in children treated with high doses of bronchodilators. Salbutamol administered by nebuliser, age more than 6 years, and hyperglycaemia were identified as risk factors of lactic acidosis during asthma. Even if self-limited, this condition is important to recognise to avoid unnecessary and harmful therapeutic intensification.

## Abbreviations

AG: Anion gap
aOR: Adjusted odds ratio
BE: Base excess
BMI: Body mass index
CI: Confidence interval
L/P: Lactate to pyruvate ratio
OR: Odds ratio
pMDI: Pressurised metered dose inhaler
SD: Standard deviations
